# Integration of bulk and single-cell transcriptomic sequencing reveals the neutrophil heterogeneity in bladder cancer and establishes a prognostic model

**DOI:** 10.1007/s12672-026-04559-3

**Published:** 2026-02-14

**Authors:** Ying-xue Song, Xiao-lin Xia, Zhi-ming Wu, Ye Yao, Jun-yu Liang, Sheng-jie Guo, Kai Yao, Hui Chang

**Affiliations:** 1 Department of Radiation Oncology, State Key Laboratory of Oncology in South China, Collaborative Innovation Center for Cancer Medicine, Sun Yat-sen University Cancer Center, Guangzhou, 510060 China; 2 Department of Oncology, Yunfu People’s Hospital, Southern Medical University, Yunfu, 527300 China; 3 Department of Urology, State Key Laboratory of Oncology in South China, Collaborative Innovation Center for Cancer Medicine, Sun Yat-sen University Cancer Center, Guangzhou, 510060 China

**Keywords:** Neutrophil, Bladder cancer, Immune infiltration, Machine learning, Single-cell RNA sequencing

## Abstract

Neutrophils are crucial immune components within the tumor microenvironment, significantly impacting tumor progression and anti-tumor immunity. To systematically characterize the heterogeneity of neutrophils in bladder cancer (BLCA), we integrated large-scale single-cell RNA sequencing (scRNA-seq) data of BLCA to define the transcriptomic landscape of neutrophil subtypes. Functional enrichment, pseudotime analysis, cell-cell communication, and deconvolution of bulk RNA sequencing (RNA-seq) samples from BLCA were conducted to comprehensively characterize the biological profiles and functions, as well as the prognostic relevance of neutrophil subtypes. A machine learning-based predictive model was developed based on the balance of prognosis-related neutrophil subtypes. We identified five distinct subtypes of neutrophils in BLCA and focused on two subtypes that were prognostically antagonistic. VEGFA+ neutrophils (Neu_0), characterized by pro-angiogenic, immunosuppressive, and extracellular matrix remodeling signatures, showed a significant correlation with poorer survival. GBP1 + neutrophils (Neu_4), characterized by response to interferon, exhibited increased innate immune activities and the production of cytokines that activate anti-tumor immunity, significantly correlated with improved survival. Pseudotime analysis positioned both Neu_0 and Neu_4 as terminal states. Cell-cell communication further identified Neu_0 as a hub orchestrating multiple pro-tumorigenic interactions. The predictive model based on the balance of Neu_0 and Neu_4 effectively stratified BLCA patients into distinct risk groups with significant differences in clinical outcomes, immune landscapes, and response profiles to antibody-drug conjugate (ADC) treatment. The investigation provided novel insights into the functional profiles of neutrophils in BLCA and offered a novel tool for guiding therapeutic strategies in BLCA.

**Supplementary Information:**

The online version contains supplementary material available at 10.1007/s12672-026-04559-3.

## Introduction

 Bladder cancer (BLCA) ranks as the ninth most common malignancy worldwide, with approximately 610,000 new cases and 220,000 deaths annually [1]. The disease represents a clinical spectrum ranging from recurrent non-muscle-invasive bladder cancer (NMIBC) requiring long-term surveillance to aggressive localized or metastatic muscle-invasive bladder cancer (MIBC) necessitating multimodal and invasive therapies. Around 10–20% of NMIBC cases further progress to MIBC, while MIBC accounts for the majority of mortality, with 5-year survival rates of below 50% and 5% for localized and metastatic diseases, respectively. Despite considerable advances in surgical and systemic therapies, clinical outcomes for BLCA are still unsatisfactory [2, 3].

The tumor microenvironment (TME) is a key determinant in the processes of tumorigenesis, progression, and therapeutic response for malignancies, including BLCA [4, 5]. The TME is dynamic and heterogeneous, consisting of diverse cell components, including tumor cells, immune cells, and stromal cells, and of extracellular matrix (ECM), vascular and lymphatic networks, and various secreted factors like growth factors, cytokines, chemokines, and extracellular vesicles. The complex interplay and balance between the anti- and pro-tumor components deeply influence the fate of tumor cells and the clinical outcome of cancer patients. Multiple critical pro-tumor mechanisms of BLCA have been reported, such as the recruitment of myeloid-derived suppressive cells (MDSCs) and regulatory T cells (Tregs), the epithelial-mesenchymal transition (EMT), the angiogenesis and lymphangiogenesis, and the IL-6/JAK/STAT3 pathway driven by the loss of SMARCB1 [6–10]. As the most abundant leukocytes in circulation and the first line of defense against abnormal host signals, neutrophils have been found to infiltrate various solid tumors extensively and have been established as a critical immune effector within the TME [11–14]. For a long time, evidence has indicated that neutrophil infiltration portends a poorer prognosis and promotes tumor progression across diverse cancer types, including BLCA [13, 15–18]. However, recent studies are recognizing the plasticity and heterogeneity of neutrophils within the TME, with some serving as a crucial component of anti-tumor immunity. In specific contexts, distinct phenotypes of neutrophils have been identified that exert anti-tumor functions through potential mechanisms, including enhancing class II antigen presentation, directly killing tumor cells, and recruiting and activating T cells and other immune effectors [19–23]. Therefore, comprehensively characterizing neutrophil heterogeneity in tumors is crucial for understanding the complexity of tumor immunity, disease progression, and prognosis, as well as for providing new insights into cancer therapy.

However, universally applicable markers for definitively distinguishing the anti- and pro-tumor phenotypes of neutrophils are still lacking across cancer types. Moreover, in BLCA, the heterogeneity and functional diversity of neutrophils remain poorly explored, leaving their specific roles in tumor immunity and disease progression unclear. While single-cell RNA sequencing (scRNA-seq) is a powerful technique to decode cellular heterogeneity, its application to neutrophils is limited by their low mRNA abundance and fragility. In this study, we integrated large-scale scRNA-seq data from BLCA tumor and urine samples, the latter in direct contact with bladder tumors and serving as representative surrogates of the BLCA TME [24]. Specifically, we employed a lowered threshold for the number of detected genes per cell during quality control to enrich for neutrophils and map their transcriptome landscape. We delineated the impact of distinct subtypes on clinical outcomes and revealed their functional programs and mechanisms of action within the BLCA TME. Additionally, we developed a machine learning-based model to assess the balance between the infiltration of pro-tumor and anti-tumor neutrophil subtypes, which could effectively predict prognosis and serve as a potential biomarker to indicate the response to antibody-drug conjugate (ADC) therapies in patients with BLCA.

## Methods

### Data sources

The scRNA-seq datasets of a total of 50 BLCA samples, including 40 tumor tissue samples and 10 urine samples from bladder tumors, were downloaded from the Gene Expression Omnibus (GEO; https://www.ncbi.nlm.nih.gov/geo/) database with the accession numbers GSE176249, GSE190888, GSE211388, GSE222315, GSE267718, and from the study by Salome et al.. (10.17632/7yb7s9769c.1) [8, 24–28]. The details of scRNA-seq samples are listed in Table S1. Bulk RNA sequencing (RNA-seq) data and clinical information of the TCGA-BLCA cohort were obtained from the UCSC Xena platform (https://xenabrowser.net/). Microarray gene expression data and clinical information for BLCA patients were obtained from the GEO database, with accession numbers GSE13507, GSE32548, GSE32894, and GSE48276, respectively [29–32].

### ScRNA-seq data processing

The Seurat (version 5.2.0) package was employed to process the scRNA-seq count matrix [33]. Genes detected in three or fewer cells were excluded from subsequent analyses when using the CreateSeuratObject function. Cells with detected genes numbered between 100 and 6000, and the percentage of mitochondrial genes below 50% were included in subsequent analysis. Doublet removal was performed by the DoubletFinder (version 2.0.4) package for each sample, with the neighborhood size (pK) set to 0.09 and the proportion of artificial doublets (pN) set to 0.25 [34]. To avoid unexpected noise and expression artefacts by dissociation, a total of 1,514 genes, which were associated with mitochondria (50 genes), heat-shock protein (178 genes), ribosome (1,253 genes), and dissociation (33 genes), and collected from the study by Xue et al., were excluded and are listed in Table S1 [35]. Then, we integrated the samples using the harmony method of Seurat, with the top 4000 highly variable genes (HVGs) and the top 20 principal components (PCs). Major cell clusters were identified with the FindClusters function (resolution = 0.1) and visualized with UMAP. We performed iterative sub-clustering on T/NK cells and the myeloid lineage to identify cell types at a more fine-grained resolution. For clustering, the top 30 PCs were selected based on the top 2000 HVGs for clustering T/NK cells (resolution = 0.3) and myeloid (resolution = 0.4). Subsequently, neutrophils were identified from the myeloid cell population and reclustered with the top 20 PCs based on the top 2000 HVGs (resolution = 0.4). Marker identification for each cluster was conducted with the FindAllMarker function, applying the threshold of min.pct > 0.1, logfc.threshold > 0.25, and adjusted *P* < 0.05 using Bonferroni correction. The clusterProfiler (version 4.14.4) package was used for Gene Ontology (GO) analysis, with a significance threshold of adjusted *P* < 0.01 using Benjamini-Hochberg (BH) correction [36].

### Pseudotime analysis

The monocle3 (version 1.3.7) package was employed to construct the pseudotime trajectory for neutrophils [37, 38]. The differentially expressed genes along the trajectory were extracted using the graph_test function, applying the threshold of q-value < 0.05 and morans_I > 0.05.

### Bulk RNA-seq deconvolution and survival analysis

The BayesPrism (version 2.2.2) package was employed for deconvolving the TCGA-BLCA bulk RNA-seq samples and inferring the infiltration levels of cell types annotated in BLCA scRNA-seq data [39]. To reduce the computational burden, we subsampled 500 cells for each subtype and generated reference profiles of the cell states. To minimize unexpected noise, we removed ribosomal protein genes, mitochondrial genes, genes located on chromosomes X and Y, and genes detected in fewer than three cells. Subsequently, protein-coding genes were selected for the BayesPrism-based deconvolution analyses of TCGA-BLCA samples.

Kaplan-Meier survival analysis was performed using the survival (version 3.8-3) package, and the resulting curves were visualized using the survminer (version 0.5.0) package. The statistical significance of the survival analysis was set at *P* < 0.05, as determined by the log-rank test. The correlation coefficients among cell infiltration proportions were calculated using the Spearman method with the cor function of the base package. The corrplot (version 0.95) package was employed for cell clustering and correlation heatmap visualization of the immune infiltration of TCGA-BLCA.

### Gene signatures quantification

Gene signature scores for neutrophils were calculated using the AUCell (version 1.28.0) package, with aucMaxRank set to 5% [40]. The MHC-I antigen presentation gene set was compiled from research by Thompson et al.. and Chen et al.., which is listed in Table S1 [41, 42]. The gene sets of remaining signatures were obtained from the hallmark gene sets and ontology gene sets of MsigDB (https://www.gsea-msigdb.org/gsea/msigdb/index.jsp). Differences were determined by the Wilcoxon test, with a significance threshold set at *P* < 0.05.

### Cell-cell communication

The CellChat (version 2.2.0) package was utilized to determine the cell-cell communication patterns [43]. To reduce computational burden, we subsampled 500 cells for each subtype to determine interaction networks. The minimum number of cells required for intercellular communication was set to 3.

### Differentially expressed genes test

The patients in the TCGA-BLCA cohort were stratified into four risk groups based on the infiltration levels of Neu_0 and Neu_4 using the median value as the cutoff: Group1 comprised samples with Neu_0 infiltration above the median and Neu_4 infiltration below the median; Group2 included samples with Neu_0 infiltration below the median and Neu_4 infiltration above the median; Group3 consisted of samples with both Neu_0 and Neu_4 infiltrations above the median; and Group4 included samples with both Neu_0 and Neu_4 infiltrations below the median.

Differentially expressed genes (DEGs) between Group1 and Group2 were identified by the DESeq2 (version 1.46.0) package [44]. The threshold for DEGs was set to adjusted *P* < 0.05 using BH correction, and |log2 fold change (FC)| > log2(1.2). Specifically, DEGs with log2FC < 0 were defined as genes upregulated in Group1, while DEGs with log2FC > 0 were defined as genes upregulated in Group2.

### Model construction and validation

We intersected the genes upregulated in Group1 with the marker genes of Neu_0 and genes upregulated in Group2 with the marker genes of Neu_4, respectively, collectively constituting neutrophil subtype-related genes (NSRGs). The prognosis-related NSRGs were identified by the Univariate Cox regression analysis with a significance threshold of *P* < 0.05 for overall survival (OS) in the TCGA-BLCA cohort. Prognosis-related NSRGs were further selected by LASSO-Cox regression analysis conducted by the glmnet (version 4.1-8) package [45]. The 10-fold cross-validation was performed to determine the optimal λ value, which corresponded to the minimum mean cross-validated error. The LASSO score was utilized as the riskscore. The riskscore was calculated using the following formula: riskscore = LASSO score of TCGA-BLCA = exp1β1 + exp2β2 + … + expi*βi. Based on the median value of riskscore, the BLCA patients included in both training and testing datasets were divided into high- and low-riskscore groups in each cohort. The independent prognostic value of the riskcore alongside the other clinical factors was evaluated by Univariate and multivariate Cox regression analyses in the TCGA-BLCA cohort. Tumor immune dysfunction and exclusion (TIDE) score of TCGA-BLCA was calculated online (http://tide.dfci.harvard.edu/) [46]. Tumor inflammation signature (TIS) score of TCGA-BLAC was calculated by GSVA with 18 genes listed in Table S1 by the GSVA (version 2.0.4) package [47, 48]. The predictive accuracy of the established prognostic model, immune cell, and immune indicator scores, including TIDE and TIS, was assessed by time-dependent receiver operating characteristic (ROC) curves with the timeROC (version 0.4) package. The correlation significance between the riskscore and immune cells was assessed using the Spearman test, with a threshold of *P* < 0.05.

### ADC response prediction

We obtained the list of genes implicated in ADC sensitivity from the study by Bosi et al., which compiled the genes related to the sensitivity and resistance to ADC from the reported research. We further selected genes overexpressed in BLCA (log2FC > 1) from their pan-cancer analysis [49]. These genes are listed in Table S1 and were utilized to derive the predicted sensitivity score by GSVA for each tumor sample of TCGA-BLCA. The correlation significance between this score and the riskscore was assessed using the Spearman method. Differences in mRNA expression levels of ADC targets between risk groups were determined by the Wilcoxon test, with a significance threshold set at *P* < 0.05.

### Statistical analysis and visualization

The statistical tests and data visualization of this work were performed with R (version 4.4.2). The relationship between P values and asterisks was as follows: **** for *P* < 0.0001 or adjusted *P* < 0.0001, *** for *P* < 0.001 or adjusted *P* < 0.001, ** for *P* < 0.01 or adjusted *P* < 0.01, and * for *P* < 0.05 or adjusted *P* < 0.05.

## Results

### The single-cell transcriptomic landscape in human BLCA

**Fig. 1:**
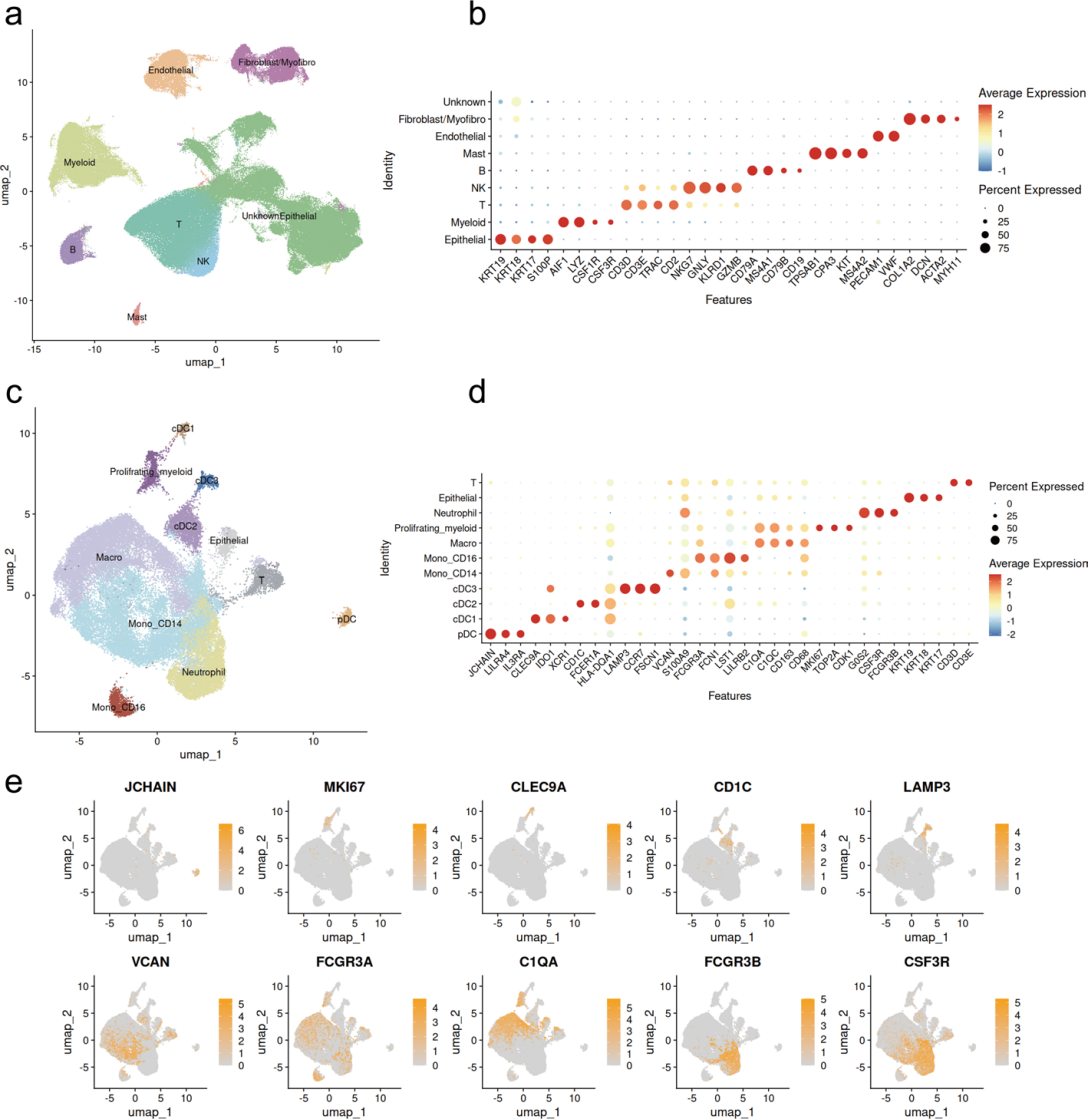
Identification of major cell types and myeloid subtypes of BLCA. **a** UMAP plot of all cells grouped by major cell types. **b** Dot plot showing the marker genes used to annotate major cell types. **c** UMAP plot of myeloid cells grouped by subtypes. **d** and **e** Dot plot and feature plot showing the marker genes used to annotate the myeloid cell subtypes

We obtained the scRNA-seq transcriptomic datasets of human BLCA from the GEO database and from the study by Salome et al. [8, 24–28]. Following quality control and batch effect correction, 290,589 cells from the tumor and urine of BLCA were retained and clustered into eight major clusters in the first round of clustering (Fig. 1a and b, and Fig. S1a and b are given in Supplement figure). We used classic markers described in the literature to annotate cell types [8, 24, 50–52]: epithelial cells (KRT19+, KRT18+); myeloid cells (AIF1+, LYZ+); B cells (CD79A+, MS4A1+); mast cells (TPSAB1+, KIT+); endothelial cells (PECAM+, VWF+); fibroblast and myofibroblast cells (COL1A2+, ACTA2+). The cluster comprising T cells and natural killer (NK) cells was further subdivided into T cells (CD3D+, CD3E+) and NK cells (NKG7+, GNLY+) during the second round of clustering (Fig. S1c and d are given in Supplement figure). The cell cluster devoid of specific markers was classified as an unknown cell type.

A total of 41,493 myeloid cells (AIF+, LYZ+) were subsequently sub-clustered, which identified nine distinct cell subtypes based on canonical markers (Fig. 1c and d) [12, 51, 53, 54], including macrophages (Macro; CD163+, CD68+); proliferating myeloid cells (MKI67+, TOP2A+); CD14 + monocytes (Mono_CD14; VCAN+, S100A9+); CD16 + monocytes (Mono_CD16; FCGR3A+, LST1+); neutrophils (FCGR3B+, CSF3R+); plasmacytoid dendritic cells (pDC; LILRA4+, IL3RA+); conventional dendritic cell type 1 (cDC1; CLEC9A+, IDO1+); conventional dendritic cell type 2 (cDC2; CD1C+, FCER1A+); and conventional dendritic cell type 3 (cDC3; LAMP3+, CCR7+). Additionally, a small number of concomitant T cells and epithelial cells were excluded from downstream analyses. Neutrophils exhibited lower mRNA abundance compared to other myeloid subtypes in our study, consistent with their known biological features as reported in prior research (Fig. S1e is given in Supplement figure) [12, 55].

### The heterogeneity of neutrophils in BLCA

**Fig. 2:**
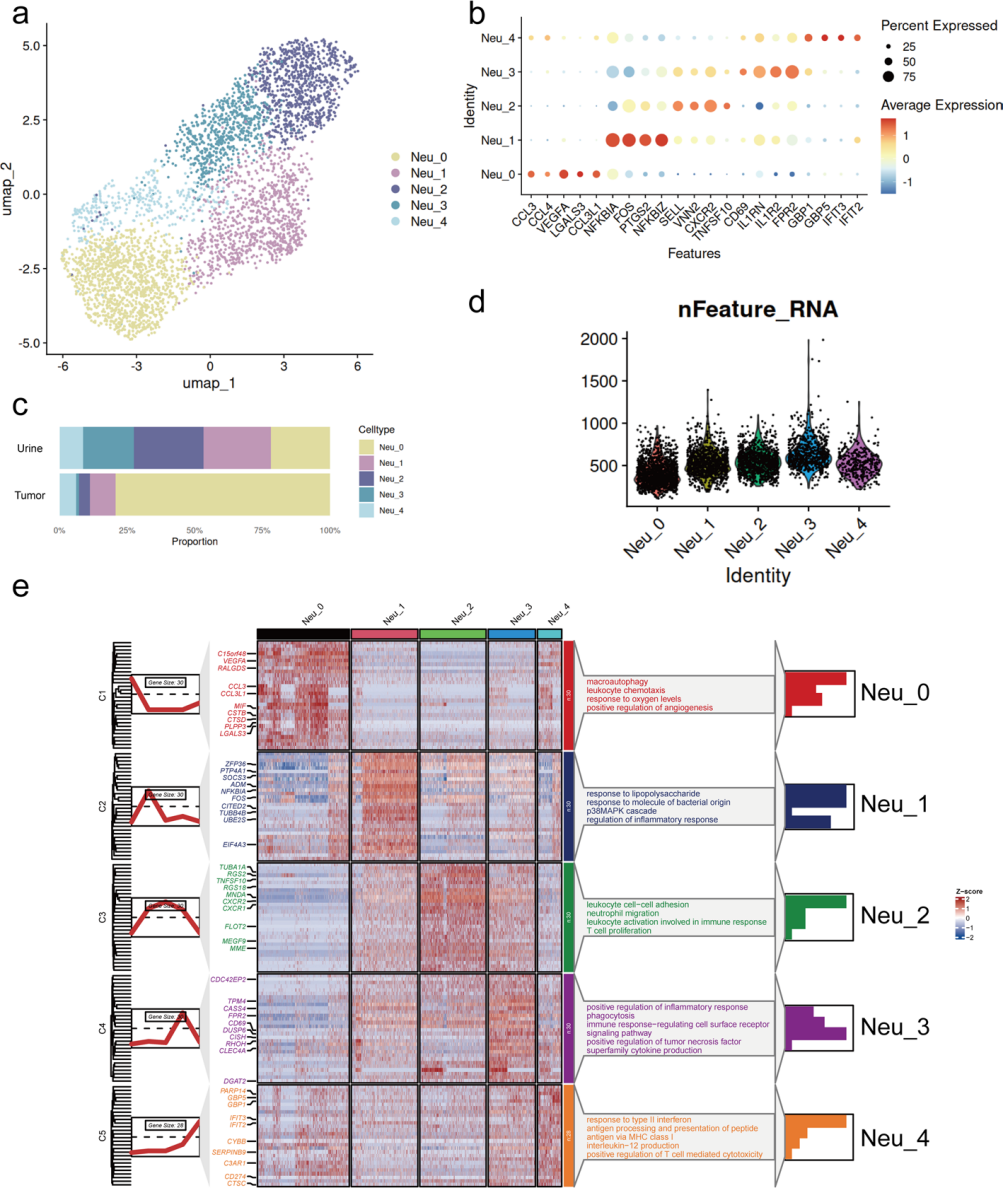
Identification of five distinct neutrophil subtypes in BLCA. **a** UMAP plot of neutrophils grouped by subtypes. **b** Dot plot showing the marker genes of neutrophil subtypes. **c** Stacked bar plots of the proportional abundance of neutrophil subtypes in tumor tissue and urine samples. **d** Violin plot showing the number of detected genes (nFeature) per cell across neutrophil subtypes. **e** Heatmap of DEGs of five neutrophil subtypes and GO enriched pathway

We extracted a total of 4,021 neutrophils (CSF3R+, FCGR3B+) from the myeloid cells and performed a third-round sub-clustering. Five distinct transcriptomic subtypes were identified as follows (Fig. 2a, b, and e): Neu_0 (VEGFA+) exhibited enrichment in positive regulation of angiogenesis and response to oxygen level, and highly expressed the pro-angiogenic factor VEGFA [56], suggesting that Neu_0 represented a distinct pro-angiogenic cell state. Furthermore, we observed the co-expression of the pro-metastatic factor LGALS3 [57], indicating the pro-tumor identity of Neu_0. Neu_1 (NFKBIZ+) was enriched in response to lipopolysaccharide and molecules of bacterial origin and exhibited high expression of inflammation-related transcription factors, NFKBIZ, which is commonly induced by lipopolysaccharide and participates in the inflammatory response and mediates inflammatory responses [58, 59]. Neu_2 (SELL+) was associated with leukocyte cell-cell adhesion, neutrophil migration, and the highly expressed SELL and VNN2, which are markers related to the transendothelial migration of neutrophils [60, 61]. Furthermore, the co-expression of the chemokine receptor, CXCR2, indicated that Neu_2 was involved in the recruitment and trafficking of neutrophils in the tumor. Neu_3 (IL1RN+) was preferentially enriched in pathways associated with phagocytosis and inflammation, such as positive regulation of inflammatory response and positive regulation of tumor necrosis factor superfamily cytokine production. IL1RN, a marker of neutrophil activation [62, 63], was observed to be highly expressed in Neu_3, suggesting the activation state of the population. Neu_4 (GBP1+) displayed high expression of genes involved in response to type II interferon, while concurrently activating pathways related to immune activation, including antigen processing and presentation of peptide antigen via MHC class I and interleukin-12 (IL-12) production. The cell state was characterized by interferon-stimulated genes (ISGs), including GBP1, the marker related to IFN-α/β/IFN-γ/IL-15 pathway, and associated with the favorable outcomes of diverse tumors [64, 65]. Neu_4 could be recognized as the neutrophil subpopulation that responded to interferon and was related to the immune activation. All five neutrophil subtypes were present across tissue types, with Neu_0 accounting for the majority in tumor tissues, while Neu_1, Neu_2, and Neu_3 were more abundant in urine samples. Neu_4 exhibited comparable proportions in both tissue types (Fig. 2c). Notably, despite the generally low mRNA content typical of neutrophils, all five subtypes exhibited comparable and stable mRNA levels, supporting their transcriptomic stability and biological relevance rather than representing low-quality artefacts (Fig. 2d).

### Pseudotime analysis revealed the differentiation trajectory of neutrophils

**Fig. 3:**
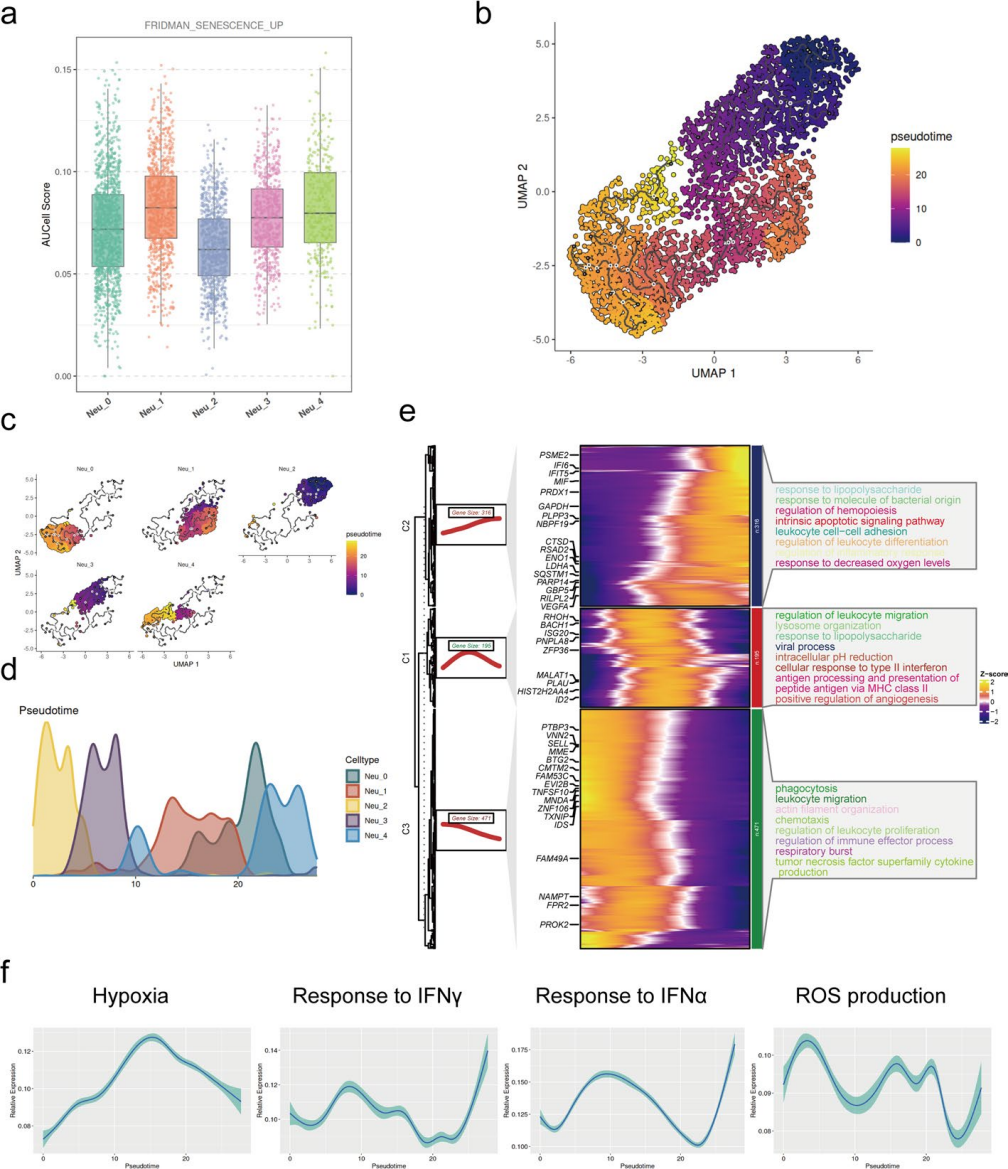
Pseudotemporal analysis of neutrophils. **a** Box plot of senescence scores across neutrophil subtypes scored by AUCell. **b** and **c** UMAP plots of neutrophil differentiation colored by pseudotime. **d** Density plot of neutrophil distribution along pseudotime. **e** Heatmap of gene expression dynamics along pseudotime. The genes were clustered hierarchically into three clusters, and the GO enriched pathways were shown. **f** Line plots of the trend of hypoxia, response to IFNγ, response to IFNα, and ROS production over pseudotime

Monocle3 was employed to construct the pseudotime trajectory of neutrophil subtypes on the UMAP of neutrophils generated by Seurat. Neu_2, characterized by SELL and CXCR2, has been described as a mature-primed and plastic state in earlier research [12, 66] and exhibited the lowest senescence score among all subtypes (Fig. 3a). Accordingly, we set Neu_2 as the trajectory root ) (Fig. 3b and c).The inferred trajectory passed through Neu_3 as an intermediate state and ultimately transitioned into three terminal cell states: Neu_0, Neu_1, and Neu_4. Along the pseudotime trajectory, Neu_2 and Neu_3 were predominantly located in the earlier stages, while Neu_0, Neu_1, and Neu_4 were enriched in the later stages (Fig. 3d).

Genes that varied significantly along the pseudotime trajectory were identified and grouped into three distinct modules based on their dynamic expression patterns (Fig. 3e). The GO enriched analysis indicated that neutrophils initially engaged in functions related to migration, chemotaxis, and production of reactive oxygen species (ROS). Over time, their roles transitioned into antigen presentation, antimicrobial function, and angiogenesis, while concurrently exhibiting increased characteristics associated with hypoxia and the response to interferon, as well as decreased characteristics of ROS production in the later stage (Fig. 3e and f).

### Prognostic significance of neutrophil subtypes in BLCA

**Fig. 4:**
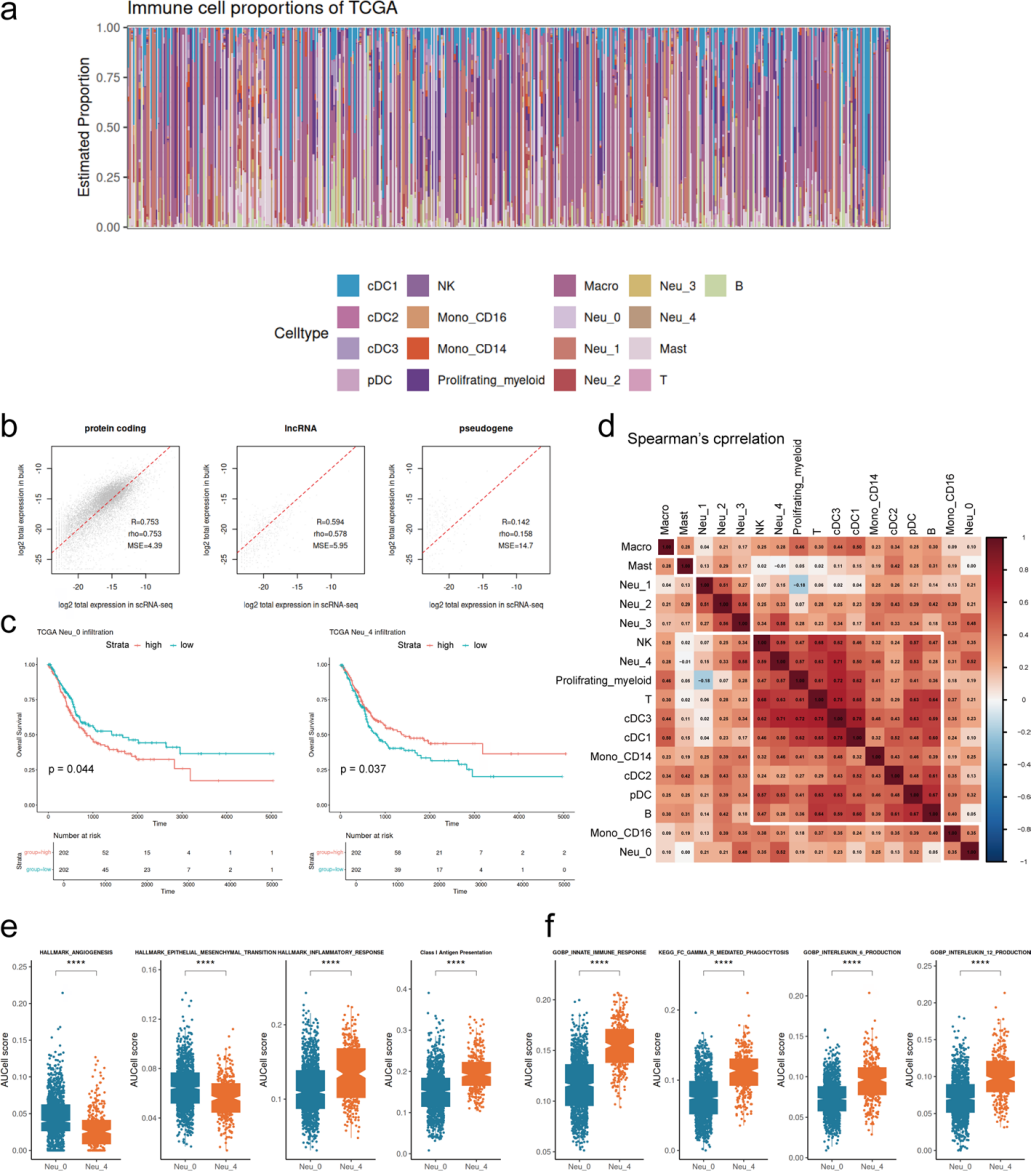
Impact of neutrophil subtypes on BLCA prognosis. **a** Stacked bar plots of immune cell infiltration proportions in the TCGA-BLCA cohort, inferred by BayesPrism. **b** Correlation of gene expression between bulk RNA-seq and scRNA-seq. **c** Kaplan-Meier survival curves of Neu_0 (left) and Neu_4 (right) infiltration in the TCGA-BLCA cohort. **d** Heatmap of Spearman’s correlation coefficients for immune cell infiltration in bulk RNA-seq samples. **f** Box plots of pathway activities between Neu_0 and Neu_4 scored by AUCell. (*P < 0.05, **P < 0.01, ***P < 0.001, ****P < 0.0001)

Bulk RNA-seq transcriptomic data of TCGA-BLCA were deconvoluted using the annotated BLCA scRNA-seq data as a reference based on the BayesPrism algorithm, thereby inferring the infiltration levels of each neutrophil subtype within individual samples (Fig. 4a and b) [39]. The immune infiltration landscape of samples in the TCGA-BLCA is illustrated in Fig. 4a. Kaplan-Meier survival analysis demonstrated that higher infiltration of Neu_0 was significantly associated with poor OS (*P* = 0.044). Conversely, higher infiltration of Neu_4 was significantly associated with improved OS of patients in this cohort (*P* = 0.037). Infiltration levels of other neutrophil subtypes, however, showed no significant association with patients’ prognosis (Fig. 4c, and Fig. S1f, g, and h are given in Supplement figure). The findings indicated that the infiltration levels of different neutrophil subtypes served as prognostic indicators, distinguishing distinct outcomes for BLCA patients.

We performed hierarchy clustering on immune cell infiltration proportions in the TCGA-BLCA cohort (Fig. 4d). Neu_4 co-clustered with innate immune cells, such as DC and NK cells with anti-tumor activity, as well as adaptive immune cells, including T and B cells, whereas Neu_0 was clustered with CD16 + monocytes. Pathways related to immune characteristics and tumor progression were further compared between Neu_0 and Neu_4 using AUCell (Fig. 4e and f). The pro-tumor signatures, including angiogenesis and epithelial-mesenchymal transition, showed higher enrichment scores in Neu_0. In contrast, the inflammatory response pathways and anti-tumor signatures, including MHC-I antigen presentation, innate immune response, Fc-γ mediated phagocytosis, and interleukin-6 (IL-6) and IL-12 production, exhibited higher expression levels in Neu_4. Collectively, distinct neutrophil subtypes exhibited heterogeneous immune features, and bladder tumors dominated by different subtypes also displayed different immune infiltration patterns.

### Cell-cell communication analysis revealing neutrophils’ interactions with other cells

**Fig. 5:**
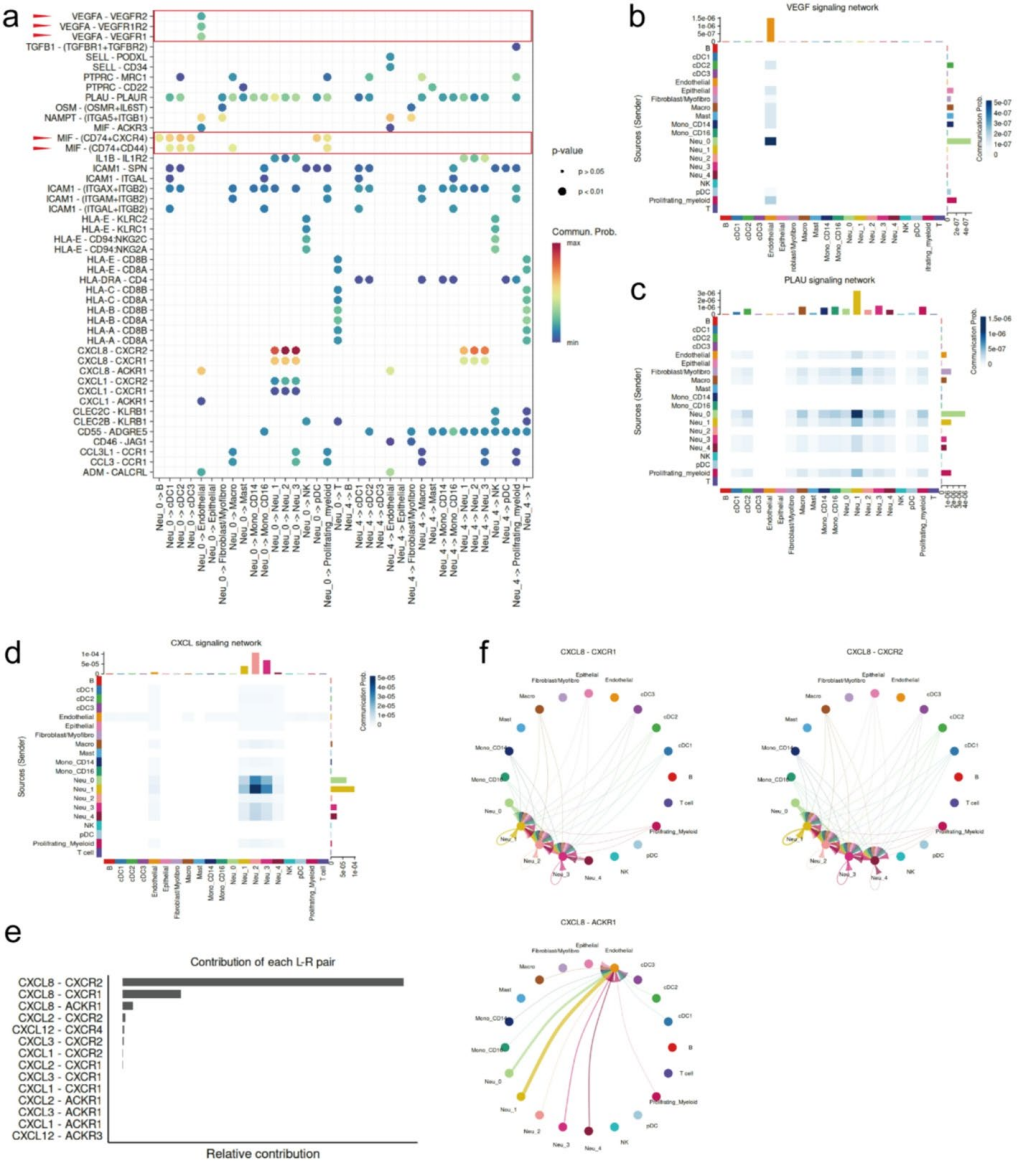
Neu_0 and Neu_4 interacted with other subtypes. **a** Bubble plot showing interactions mediated by ligands from Neu_0 and Neu_4 with receptors on other cells. **b**, **c**, and **d** Heatmaps of interaction strength of VEGF, PLAU, and CXCL ligand-receptor pathways in the TME. **e** Bar plot of the relative contributions of individual CXCL ligand-receptor pairs. **f** Circle plots of CXCL8 interaction network with its receptor pairs

To further investigate the interactions between neutrophils and other cell types within tumors, we performed CellChat to analyze the receptor-ligand pathways in BLCA. Neutrophils exhibited a greater number of ligands corresponding to receptors on cDCs, endothelial cells, macrophages, CD16 + monocytes, NK cells, proliferating myeloid cells, T cells, and other neutrophil subtypes (Fig. S2a is given in Supplement figure). Next, we investigated the ligand-mediated interactions of Neu_0 and Neu_4 with other cell types (Fig. 5a). Neu_0 displayed distinctive expression of VEGFA, whose receptors included VEGFR1, VEGFR2, and VEGFR1R2, expressed in endothelial cells. Notably, our data indicated that Neu_0 was the predominant source of VEGFA interacting with endothelial cells in the TME (Fig. 5b and Fig. S2b, c, d, and e are given in Supplement figure). The cytokine is a powerful driver of angiogenesis associated with a poorer prognosis in BLCA [56], and we pinpointed its main dominance to Neu_0 in BLCA. Compared to Neu_4, Neu_0 exhibited stronger expression of MIF and specific interactions with B cells, cDCs, macrophages, pDCs, and proliferating myeloid cells through MIF-CD74 + CXCR4 or MIF-CD74 + CD44. The MIF-CD74 axis was reported to facilitate the immunosuppression and metastasis of tumors, which may contribute to the immunosuppression activity of Neu_0 [67]. Additionally, in outgoing ligand pathways, Neu_0 was a prominent source of PLAU (uPA) (Fig. 5c), a protease that could degrade ECM, facilitating the dissemination of tumor cells. Furthermore, our data indicated that the PLAU derived from the Neu_0 interacted with PLAUR (uPAR) expressed in multiple cell types within the BLCA TME, including fibroblasts and macrophages. This interaction was reported to further engage in ECM remodeling and immune suppression in the TME and was significantly associated with tumor progression and poorer survival outcomes [68–71]. Our findings established a mechanistic link between the Neu_0 subtype and the pro-tumor activity mediated by PLAU.

**Fig. 6:**
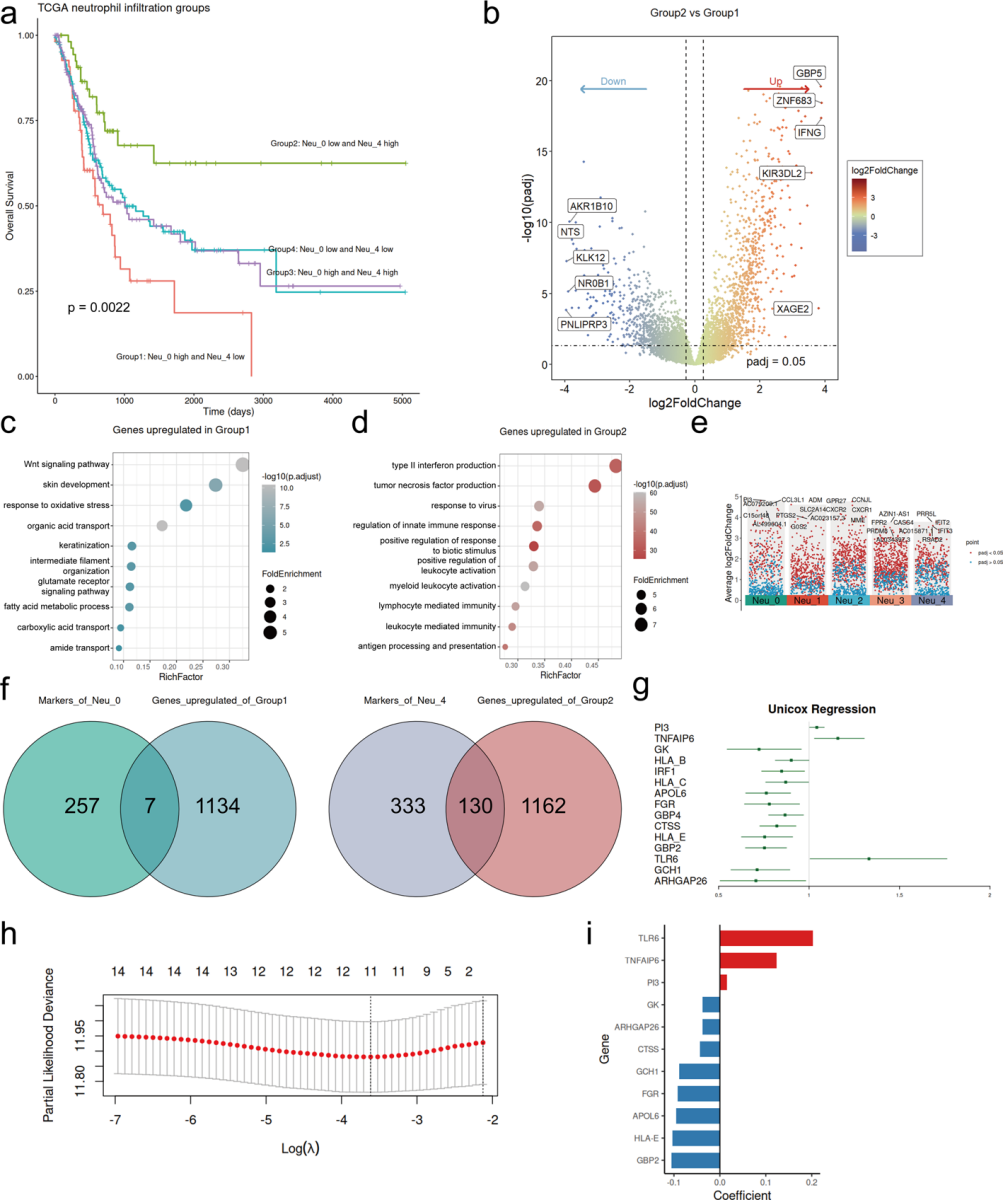
Development of a neutrophil-subtype-based prognostic model for BLCA with machine learning. **a** Kaplan-Meier survival curves of four groups stratified by median infiltration of Neu_0 and Neu_4. **b** Volcano plot of DEGs between Group1 and Group2. **c** and **d** Bubble plots of GO enriched pathways for genes upregulated in Group1 and Group2, respectively. **e** Volcano plot of marker genes of neutrophil subtypes. **f** Venn plots of gene intersections for the identification of NSRGs. **g** Forest plot of prognosis-associated NSRGs identified from univariable Cox regression analysis (*P* < 0.05). **h** Cross-validation for the LASSO-Cox regression model to select the tuning parameter. **i** Bar plot of regression coefficients of 11 NSRGs in the LASSO-Cox regression model

Furthermore, neutrophils were identified as major sources of CXCL cytokines within the TME (Fig. 5d). CXCL8, produced predominantly by Neu_0 and Neu_1, constituted a major component of the CXCL ligand pathway (Fig. 5e) and further enhanced the recruitment of additional neutrophils into the bladder tumor via the CXCL8-CXCR1 and CXCL8-CXCR2 axes (Fig. 6f). Furthermore, Neu_0 and Neu_1 interacted with endothelial cells via the CXCL8-ACKR1 axis, which could enhance angiogenesis in the tumor (Fig. 5f) [72].

In summary, the cell communication analysis revealed that neutrophils engaged in complex interactions with other cell types. Neu_0 exhibited a greater presence of pro-tumor ligands in its interactions compared to Neu_4 and acted as a crucial regulator coordinating multiple signaling pathways to facilitate tumor progression.

### Development of a prognostic model associated with neutrophils

Based on different infiltration patterns of prognostically relevant neutrophil subpopulations, the patients in the TCGA-BLCA were categorized into four groups with the median value of the infiltration level of Neu_0 and Neu_4 as the cutoff point. Significant differences in prognosis among the groups were demonstrated by Kaplan-Meier survival analysis (Fig. 6a). Patients in Group1, with high levels of Neu_0 and low levels of Neu_4, had the poorest survival. Conversely, Patients in Group2, with high levels of Neu_4 and low levels of Neu_0, exhibited the best survival. We further identified 2296 DEGs between Group1 and Group2 (Fig. 6b). The genes upregulated in Group1 were enriched in pathways included in metabolism, epithelial development, and the Wnt signaling pathway (Fig .6c). The genes upregulated in Group2 were enriched in immune activation and cytokine production, including the regulation of innate immune response, response to biotic stimulus and virus, and the production of type II interferon and tumor necrosis factor (Fig .6d). This analysis revealed different neutrophil subtype infiltration patterns characterizing distinct TMEs, which had implications for tumor behavior and patient prognosis.

**Fig. 7:**
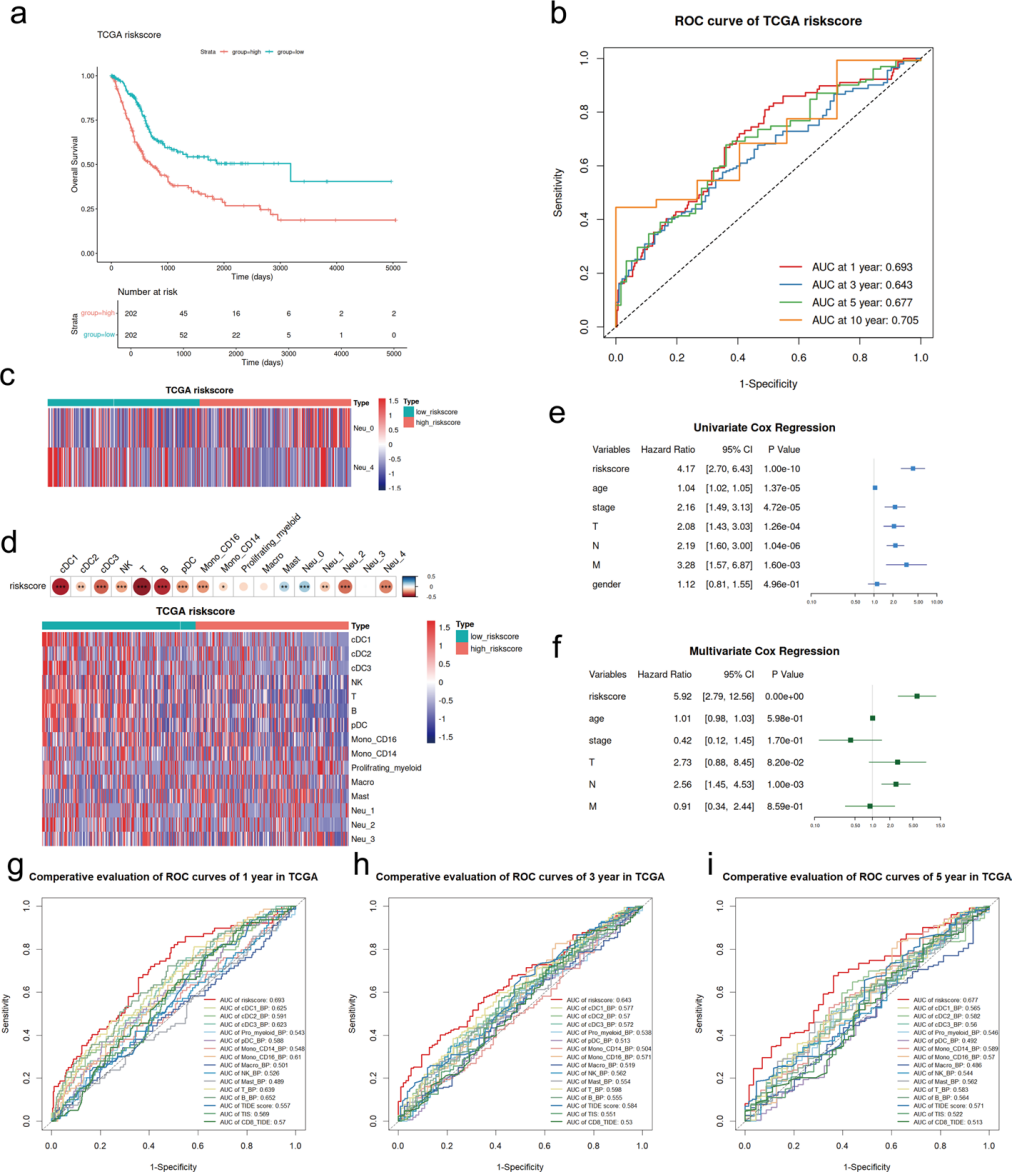
Validation of the neutrophil subtype-based prognostic model in the TCGA-BLCA cohort. **a** Kaplan-Meier survival curve comparing OS between high-riskscore and low-riskscore groups. **b** Time-dependent ROC curves illustrating the predictive performance of the prognostic model for 1-, 3-, 5-, and 10-year survival in the TCGA-BLCA cohort. **c** Heatmap of Neu_0 and Neu_4 infiltration. **d** Correlation between the riskscore and immune cell infiltration. (Upper) bubble plot of Spearman’s correlation coefficients (*P < 0.05, **P < 0.01, ***P < 0.001). (Lower) heatmap of other immune cell infiltration. **e** Forest plot of univariate Cox regression analysis for the riskscore and clinical factors. **f** Forest plot of multivariate Cox regression analysis for the riskscore and clinical factors. **g**, **i**, and **h** Comparative time-dependent ROC curves of riskscore, immune cells, and immune indicators for 1-, 3-, and 5-year survival in the TCGA-BLCA cohort

Subsequently, we utilized the TCGA-BLCA cohort as the training dataset to develop a prognostic model based on neutrophil infiltration patterns. We intersected the marker genes of Neu_0 with the genes upregulated in Group1 and the marker genes of Neu_4 with the genes upregulated in Group2, identifying a total of 137 NSRGs (Fig. 6e and f). Univariate Cox regression analysis further identified 15 genes among the NSRGs that were significantly associated with OS (*P* < 0.05) (Fig. 6g). Based on the 15 prognosis-related NSRGs selected from the univariate Cox regression, we further established a prognostic risk model through LASSO-Cox regression by selecting minimum mean cross-validated error of λ and defined the LASSO score as the riskscore (Fig. 6h). The formula of the riskscore based on 11 key genes, including TLR6, TNFAIP6, PI3, GK, ARHGAP26, CTSS, GCH1, FGR, APOL6, HLA-E, GBP2, whose coefficients are listed in Table S1. The patients from the TCGA-BLCA cohort were stratified into two groups based on the median value of their riskscores (Fig. 6i). The patients in the high-riskscore group exhibited poorer survival (*P* < 0.0001) in the training cohort (Fig. 7a). The performance of the risk model was further evaluated with time-dependent ROC, and the areas under the curves (AUCs) of 1-, 3-, 5- and 10-year survival rates in the training dataset were 0.693, 0.643, 0.677 and 0.701, respectively (Fig. 7b). The riskscore functioned as an independent prognostic factor in both univariate Cox regression (*P* < 0.0001, hazard ratio (HR) = 4.17, 95% confidence interval (CI) = 2.70–6.43) and multivariate Cox regression (*P* < 0.0001, HR = 5.92, 95%CI = 2.79–12.56) (Fig. 7e and f). Patients with higher riskscores exhibited increased infiltration of Neu_0 and decreased infiltration of Neu_4 (Fig. 7c). Additionally, the riskscore demonstrated significant negative correlations with the infiltration levels of cDCs, NK cells, T cells, B cells, pDCs, monocytes, and both Neu_1 and Neu_2 subtypes (*P* < 0.05) (Fig. 7d). Furthermore, the AUCs showed that the riskscore had the best performance compared to other immune cell infiltration levels and immune indicators, including TIDE and TIS, at 1-, 3-, and 5-year follow-ups in the TCGA-BLCA cohort (Fig. 7g, h, and i).

**Fig. 8:**
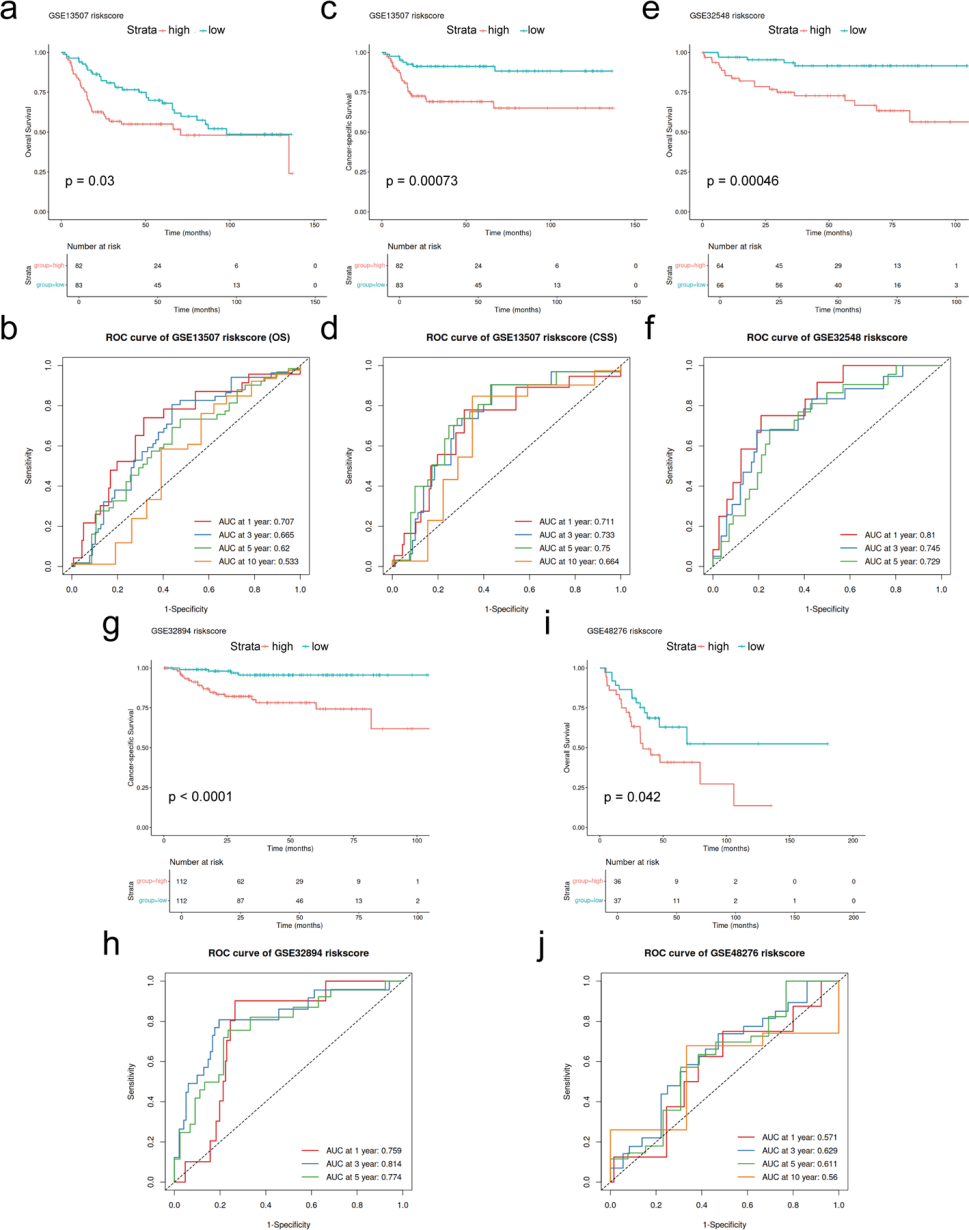
Validation of the neutrophil subtype-based prognostic model in external cohorts. **a**, **c**, **e**, **g**, and **i** Kaplan-Meier survival curves comparing OS or CSS between high-riskscore and low-riskscore groups in the GEO datasets (GSE13507, GSE32548, GSE32894, GSE48276). **b**, **d**, **f**, **h**, and **j** Time-dependent ROC curves of the predictive performance of the prognostic model for 1-, 3-, 5-, and 10-year survival in the corresponding cohorts

To further assess the generalization and efficacy of the model based on Neu_0 and Neu_4 in predicting the prognosis of patients with BLCA, four distinct GEO datasets of BLCA (GSE13507, GSE32548, GSE32894, and GSE48276) were used for the external validation (Fig. 8). The riskscores for patients in the validation datasets were calculated using the LASSO score formula developed from the TCGA-BLCA training dataset. Patients within each cohort were stratified into high-riskscore and low-riskscore groups based on the median value cutoff of the datasets. The survival outcomes exhibited significant differences between riskcore groups in these independent external validations consistently, with patients in high-riskscore groups exhibiting poor survival and in low-riskscore groups exhibiting increased survival (all *P* < 0.05). The AUCs for the predictive model of 1-year, 3-year, and 5-year survival rates were 0.711, 0.733, and 0.750 for cancer-specific survival (CSS) and 0.707, 0.665, and 0.620 for OS, respectively, in the GSE13507 dataset; 0.810, 0.745, and 0.729 for OS, respectively, in the GSE32548 dataset; 0.759, 0.814, and 0.774 for CSS, respectively, in the GSE32894 dataset; 0.571, 0.629, and 0.611 for OS, respectively, in the GSE48276 dataset. The validation across the external cohorts indicated the generalization and effective risk-stratification capabilities of the model based on the Neu_0 and Neu_4 in predicting the prognosis of BLCA patients.

### The potential benefit of ADC therapy in different risk groups

**Fig. 9:**
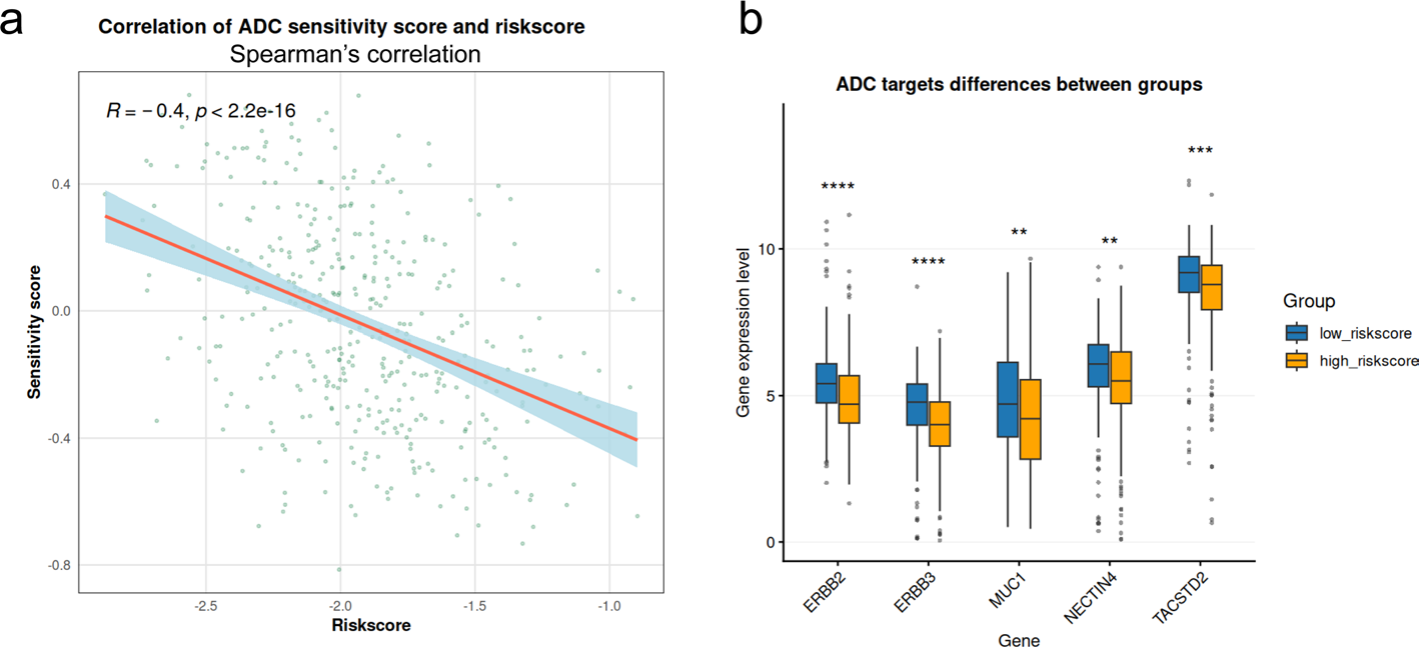
Prediction of ADC response between risk groups of TCGA-BLCA. **a** The Spearman’s correlation analysis of riskscore and sensitivity scores. **b** The mRNA expression levels of ADC targets between risk groups. (* for *P* < 0.05, ** for *P* < 0.01, *** for *P* < 0.001, **** for *P* < 0.0001)

**Fig. 10:**
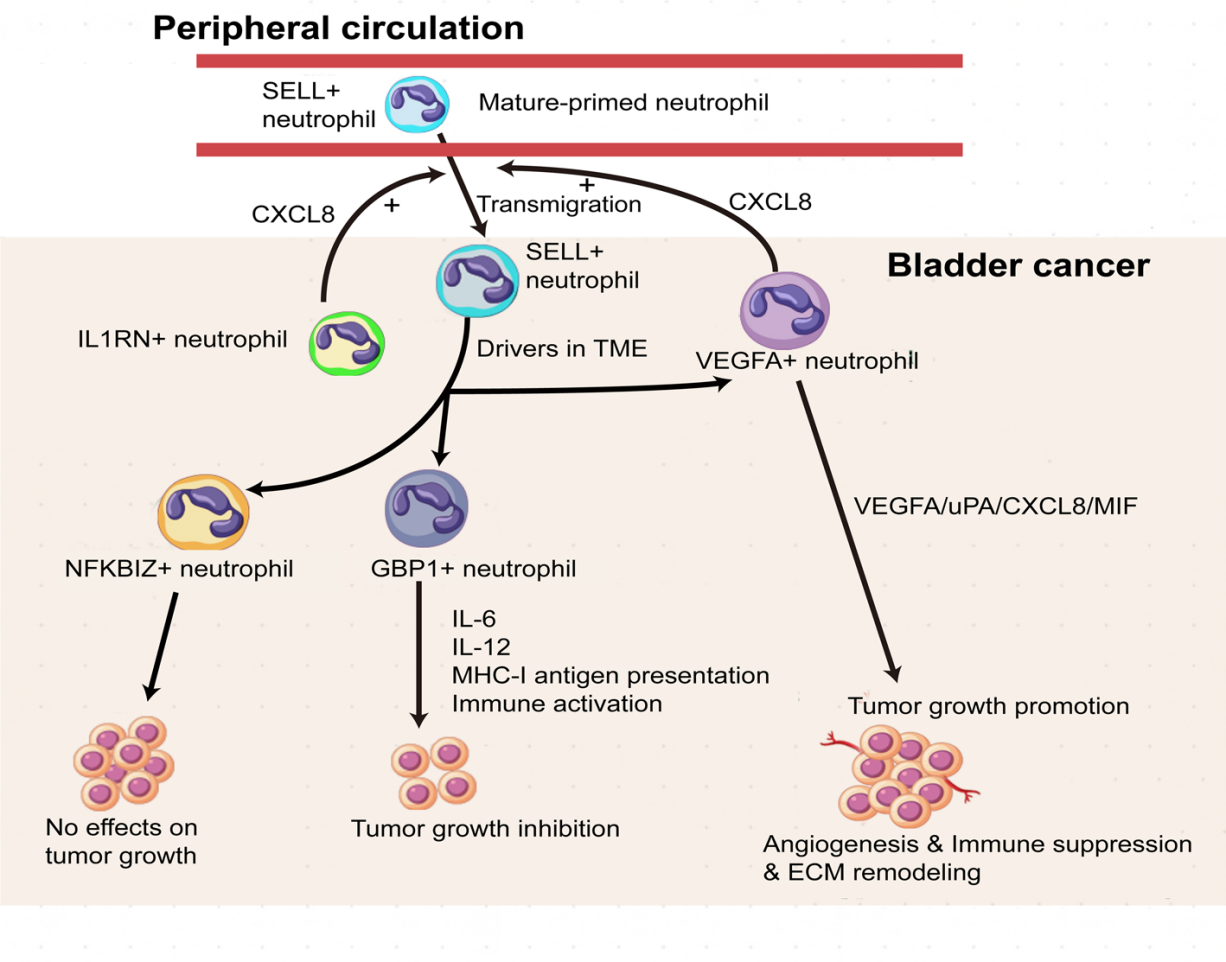
Neutrophil subtypes and their inferred functional roles in BLCA.

To access the potential ADC therapy response in the TCGA-BLCA cohort, we performed GSVA using the genes reported to associate with ADC sensitivity to generate the corresponding sensitivity score [49]. A significant positive correlation was observed between the riskscore and the sensitivity score (*R* = − 0.4, *P* < 2.2e-16) (Fig. 9a). Furthermore, we compared the expression levels of the key ADC targets between different risk groups (Fig. 9b). The samples in the low-riskscore group displayed significantly higher mRNA expression of relevant targets, including ERBB2, ERBB3, MUC1, NECTIN4, and TACSTD2 (all *P* < 0.01). Collectively, our findings demonstrated that our neutrophil-subtype-based risk model served as a potential biomarker for predicting favorable responses to ADC therapies, suggesting that BLCA patients with lower riskscores were more likely to benefit from such therapies (Fig. 10).

## Discussion

The classic activation phenotypes of tumor-associated neutrophils include anti-tumorigenic (N1) and pro-tumorigenic (N2) states established from murine models [73]. However, the actual states of neutrophils in the TME have been proven to exhibit greater heterogeneity and functional plasticity than the binary classification diagram [12]. In human BLCA, we integrated large-scale scRNA-seq data and identified five distinct neutrophil subtypes. These subtypes were marked by VEGFA (Neu_0), NFKBIZ (Neu_1), SELL (Neu_2), IL1RN (Neu_3), and GBP1 (Neu_4), respectively. The transcriptomic features of these neutrophil subtypes in our data corresponded with previously published states on benign or malignant conditions, and all five subtypes exhibited comparable and stable mRNA abundance, further validating the robustness of the transcriptome of each subtype identified in our study [35, 66, 74]. Among these states of neutrophils in BLCA, Neu_0 is characterized by a pro-angiogenic transcriptomic profile with high expression of VEGFA, overlapping with part of the pro-tumorigenic mechanisms reported for N1 phenotype in murine models [73]. For the remaining four states, however, there is no consensus on their designation as N1 or N2 in BLCA.

The trajectory analysis of neutrophils revealed a model of dynamic reprogramming, in which the mature-primed state transformed into three distinct terminal states: Neu_0, Neu_1, and Neu_4, with different functional characteristics, including angiogenesis, inflammation, and interferon response. It suggested that the fate of neutrophils migrating into the tumor was not predetermined but could be shaped by specific factors and signals in the context, allowing them to play different roles in tumors. Notably, the dominance of pro-angiogenic and immunosuppressive Neu_0 in tumor tissues indicated that the presence of the specific factors in the tumor extensively drove the phenotype of neutrophils towards a pro-tumoral fate. However, the current presence of terminal states Neu_1 and Neu_4 in tumors demonstrated that differentiation into Neu_0 was not the only termination. The dominant neutrophil subtype in the TME might be redirected by modifying key instructional signals, suggesting a potential for targeted intervention to shift the phenotypic balance in tumors.

Through the integration of bulk RNA-seq and scRNA-seq data using BayesPrism, we inferred the infiltration levels of annotated cell subtypes in tumor samples from the TCGA-BLCA cohort. Neu_0 and Neu_4 were identified as significantly associated with prognosis in human BLCA. Neu_0, marked by VEGFA, was a predictor of the poorer prognosis in patients with BLCA. Intriguingly, neutrophils marked by VEGFA were also identified in human NSCLC, pancreatic ductal adenocarcinoma, and glioblastoma, where they were similarly linked with adverse outcomes [12, 66, 75, 76]. Our finding of Neu_0 in human BLCA corroborated these observations and collectively suggested the VEGFA+ neutrophils might represent a conserved subtype serving as a potent pro-tumor entity across multiple human cancer types. Conversely, Neu_4, characterized by ISGs, was significantly correlated with improved prognosis in BLCA. Previous studies have yielded varying conclusions regarding the role of interferon-responsive neutrophils in the tumor biology of murine models and in vitro experiments [22, 35, 77, 78]. In human BLCA, however, we demonstrated that interferon-responsive neutrophils could be classified as an anti-tumor neutrophil phenotype and served as a strong prognostic indicator for BLCA. Neu_4 exhibited stronger anti-tumor characteristics, including class I antigen presentation, innate immune response, and the production of IL-6 and IL-12, as well as a lower pro-angiogenic profile, compared to Neu_0. These features associated with Neu_4, which overlapped with the anti-tumor signatures of neutrophils reported in previous studies [22], suggested a possible contribution to enhancing T cell activation and modulating immune responses in BLCA. The coexistence of two neutrophil subtypes with distinct impacts on BLCA outcome mapped their divergent pro- and anti-tumor functions onto distinct, transcriptionally defined phenotypes in this malignancy.

Our cell communication analysis revealed that Neu_0 exhibited distinct interactions with other cells compared to Neu_4, specifically in the VEGFA-VEGFR, CXCL8-CXCR1/CXCR2/ACKR, and PLAU-PLAUR pathways. Early studies emphasize the key role of VEGFA in malignancy progression mediated by neutrophils through pro-angiogenesis [79–82]. Clinical observations have consistently demonstrated a correlation between neutrophil infiltration and resistance to anti-VEGF therapies [83]. In our study, semi-quantitative analysis further pinpointed the Neu_0 as the leading contributor of VEGFA in the TME of BLCA, rather than the total neutrophil population or other cell types. In addition, Neu_0 acted as one of the leading contributors of CXCL8, a recognized pro-angiogenic factor [84], underscoring the multifaceted pro-angiogenesis program of the subtype. Moreover, CXCL8 from Neu_0 further recruited more neutrophils into the tumor, amplifying the roles of the cells. In addition to promoting angiogenesis, Neu_0 also served as the primary contributor of PLAU within the TME, a factor that has been reported to remodel the ECM and facilitate immunosuppression of tumors [68–71]. The levels of PLAU were observed to be significantly correlated with the metastasis, progression, and worse outcomes of malignancy [85]. Our semi-quantitative analysis further localized the pro-tumor factor on Neu_0 in BLCA, revealing the key cellular component in the PLAU-mediated disease progression. Furthermore, Neu_0 exhibited stronger interactions with other immune cells by MIF in contrast to Neu_4, exerting immunosuppressive effects [67]. These interactions collectively established Neu_0 as a pleiotropic entity that coordinately mediated pro-angiogenesis, immunosuppression, and ECM remodeling. In contrast to Neu_0, Neu_4 did not significantly contribute to the pro-tumorigenic interactions described above, but instead engaged preferentially in communications that supported immune surveillance and defense mechanisms. Taken together, our analysis suggested that Neu_0 potentially acted as the principal contributor to the pro-tumorigenic effects mediated by tumor-infiltrating neutrophils.

To convert these findings into a practical clinical tool, we developed a prognostic model to stratify BLCA patients based on the characteristics of Neu_0 and Neu_4, two prognostically antagonistic neutrophil subtypes. The model served as a transcriptomic indicator, quantifying the dynamic balance of these two neutrophil fates within the tumor. In particular, the high-risk group exhibited a higher proportion of Neu_0, while the low-risk group showed a higher prevalence of Neu_4. The robust and generalizable prognostic performance of this model was validated across multiple independent BLCA cohorts, underscoring its clinical reliability. Additionally, patients grouped by high-riskscore and low-riskscore groups displayed distinct immune landscapes. Specifically, the low-riskscore group, characterized by the dominance of Neu_4, delineated an immune-synergistic niche characterized by the enrichment of diverse anti-tumor effectors. Conversely, the high-riskscore group, reflecting the dominance of Neu_0, delineated an immune-rejected niche, marked by the exclusion of most anti-tumor effector cells. Our model leveraged the balance between Neu_0 and Neu_4 to stratify BLCA patient prognosis, further revealing distinct immune microenvironment patterns tied to this balance. Moreover, compared to other immune cell-based stratification and tumor immune phenotype scores, our risk model exhibited a better risk-stratified performance, indicating that the subtypes or activities of neutrophils might serve as a novel biological supplementary dimension and signature extending the lymphocyte-centric classification in predicting the prognosis of BLCA patients.

Research has established a link between the unique immune landscapes of BLCA and tumor lineage plasticity, and has further shown that the lineage plasticity involved in BLCA is associated with the expression of ADC targets and potential treatment effectiveness [86, 87]. Considering the distinct immune niche between risk groups, we explored the differential mRNA expression levels of ADC targets and the association between the potential sensitivity of ADC and riskscores. Notably, we observed that patients in different risk groups, as determined by our neutrophil-subtype-based model, exhibited significant differences in both predicted ADC sensitivity and expression of ADC targets, indicating the potential benefits of patients with lower riskscores and diminished response of patients with higher riskscores. Therefore, these findings revealed that the model functioned as an integrative biomarker, linking prognostic information, tumor immune microenvironment, and predictions of potential sensitivities to ADC therapies in BLCA, which might offer an integrative tool to stratify patients for optimized treatment.

Despite the insights gained, it is important to note that our study was subject to several limitations. First, the scRNA-seq data of this study were integrated from multiple datasets, which introduced inherent heterogeneity in terms of experimental protocols, sample types, patient demographics, disease stages, and prior treatments, potentially limiting the generalizability of our findings. Second, although we employed robust bioinformatic methods for analysis of transcriptomic data, further functional validation of neutrophil subtypes and their mechanisms still requires in vitro/in vivo experimental studies. Third, the exact factor or signaling mechanism driving the neutrophil phenotypes remained unclear, necessitating further exploration in future studies. Finally, while our prognostic model demonstrated effective performance across multiple cohorts and predictive performance for ADC, its clinical utility and significance for therapy required further validation in prospective and multi-center studies.

Overall, our study demonstrated the coexistence of both anti- and pro-tumor phenotypic neutrophils within the TME of BLCA. In particular, the VEGFA+ subtype represented a pro-tumorigenic phenotype, characterized by multiple key pro-tumorigenic signatures. In contrast, the GBP1 + neutrophils displayed an anti-tumor phenotype, marked by enhanced cytokine production and immune-activating capacities (Fig. 10). We further constructed a model that characterized the balance of two prognostically related neutrophil subtypes, which could effectively stratify the risk of BLCA patients’ outcomes and distinguish them with different ADC response profiles. Notably, our work offered a new perspective on potential therapeutic strategies for neutrophils, specifically targeting Neu_0 or inhibiting the conversion of neutrophils to the Neu_0 phenotype, which could abrogate diverse pro-tumor pathways. Such a therapy may offer a safer and more effective choice compared to the global depletion of neutrophils, which preserves the anti-infection and potentially anti-tumor functions of other neutrophil subtypes. Furthermore, the patients stratified into the high-riskscore group based on our model exhibited higher infiltration levels of the pro-tumor neutrophil subtype, suggesting they potentially benefit more from the depletion of neutrophils. New insights were provided into the role of neutrophils in the prognosis and potential treatment of BLCA patients.

## Supplementary Information

Below is the link to the electronic supplementary material.

Supplementary Material 1.

Supplementary Material 2.

## Data Availability

All datasets analyzed in this study are publicly available. The scRNA-seq datasets are available from the Gene Expression Omnibus (GEO) repository on the NCBI website (https://www.ncbi.nlm.nih.gov/geo/) under accession numbers GSE176249, GSE190888, GSE211388, GSE222315, and GSE267718, as well as from the study by Salome et al., which is available in the Mendeley Data repository at https://doi.org/10.17632/7yb7s9769c.1. The bulk RNA-seq and clinical data of the TCGA-BLCA cohort are available from the UCSC Xena platform (https://xenabrowser.net/). The microarray datasets are available from the GEO repository on the NCBI website (https://www.ncbi.nlm.nih.gov/geo/) under accession numbers GSE13507, GSE32548, GSE32894, and GSE48276.

## Declarations

### Dual publication

The research presented in this manuscript has not been submitted elsewhere for publication.
