# Supplementary material for: Integration of bulk and single-cell transcriptomic sequencing reveals the neutrophil heterogeneity in bladder cancer and establishes a prognostic model
Source: Discov Oncol. 2026 Feb 14;17:329. doi: 10.1007/s12672-026-04559-3 (PMC12917091; doi:10.1007/s12672-026-04559-3)
Supplement: Supplementary file 2 — Supplementary Material 2. [file 12672_2026_4559_MOESM2_ESM.pdf]

Article title:

Integration of bulk and single-cell transcriptomic sequencing reveals the neutrophil heterogeneity in bladder cancer and establishes a prognostic model

Journal name:

Discover Oncology

Author names:

Ying-xue Song, Xiao-lin Xia, Zhi-ming Wu, Ye Yao, Jun-yu Liang, Hui Chang, Kai Yao, Sheng-jie Guo

Corresponding author:

Hui Chang, Department of Radiation Oncology, State Key Laboratory of Oncology in South China, Collaborative Innovation Center for Cancer Medicine, Sun Yat-sen University Cancer Center, Guangzhou 510060, China. Email: changhui@sysucc.org.cn

Kai Yao, Department of Urology, State Key Laboratory of Oncology in South China, Collaborative Innovation Center for Cancer Medicine, Sun Yat-sen University Cancer Center, Guangzhou 510060, China. Email: yaokai@sysucc.org.cn

Sheng-jie Guo, Department of Urology, State Key Laboratory of Oncology in South China, Collaborative Innovation Center for Cancer Medicine, Sun Yat-sen University Cancer Center, Guangzhou 510060, China. Email: guoshj@sysucc.org.cn

Fig. S1 Integration of datasets, T/NK cell characterization, myeloid cell quality control, and neutrophil subtypes survival analysis in BLCA

(**a**) UMAP plot of all cells grouped by tissue type (tumor tissue and urine samples from BLCA patients). (**b**) Stacked bar plots of the proportional abundance of major cell types in tumor tissue and urine samples. (**c**) UMAP plot of T/NK cells grouped by subtypes. (**d**) Dot plot showing the marker genes used to annotate the T and NK cell subtypes. (**e**) Violin plot showing the number of detected genes (nFeature) per cell across myeloid cell subtypes (**f**, **g,** and **h**) Kaplan-Meier survival curves of Neu_1, Neu_2, and Neu_3 infiltration in the TCGA-BLCA cohort.

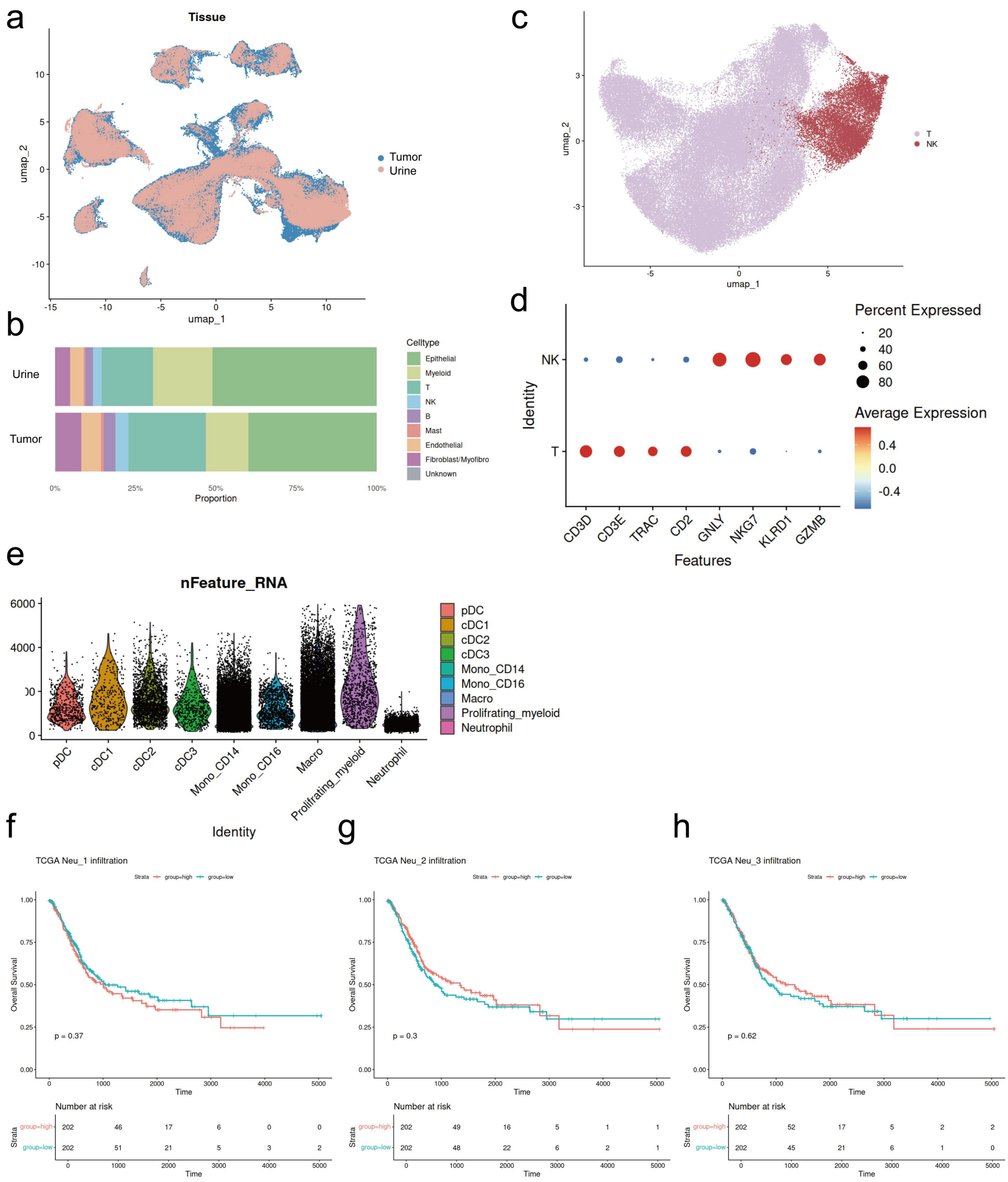

Fig. S2 Supplemental information on cell-cell communication

(**a**) Circle plots showing the number of outgoing interactions from neutrophil subtypes to other cell types. (**b**) Bar plot of the relative contributions of individual VEGF ligand-receptor pairs. (**c**, **d**, and **e**) Circle plots of the VEGFA interaction network with its receptor pairs.

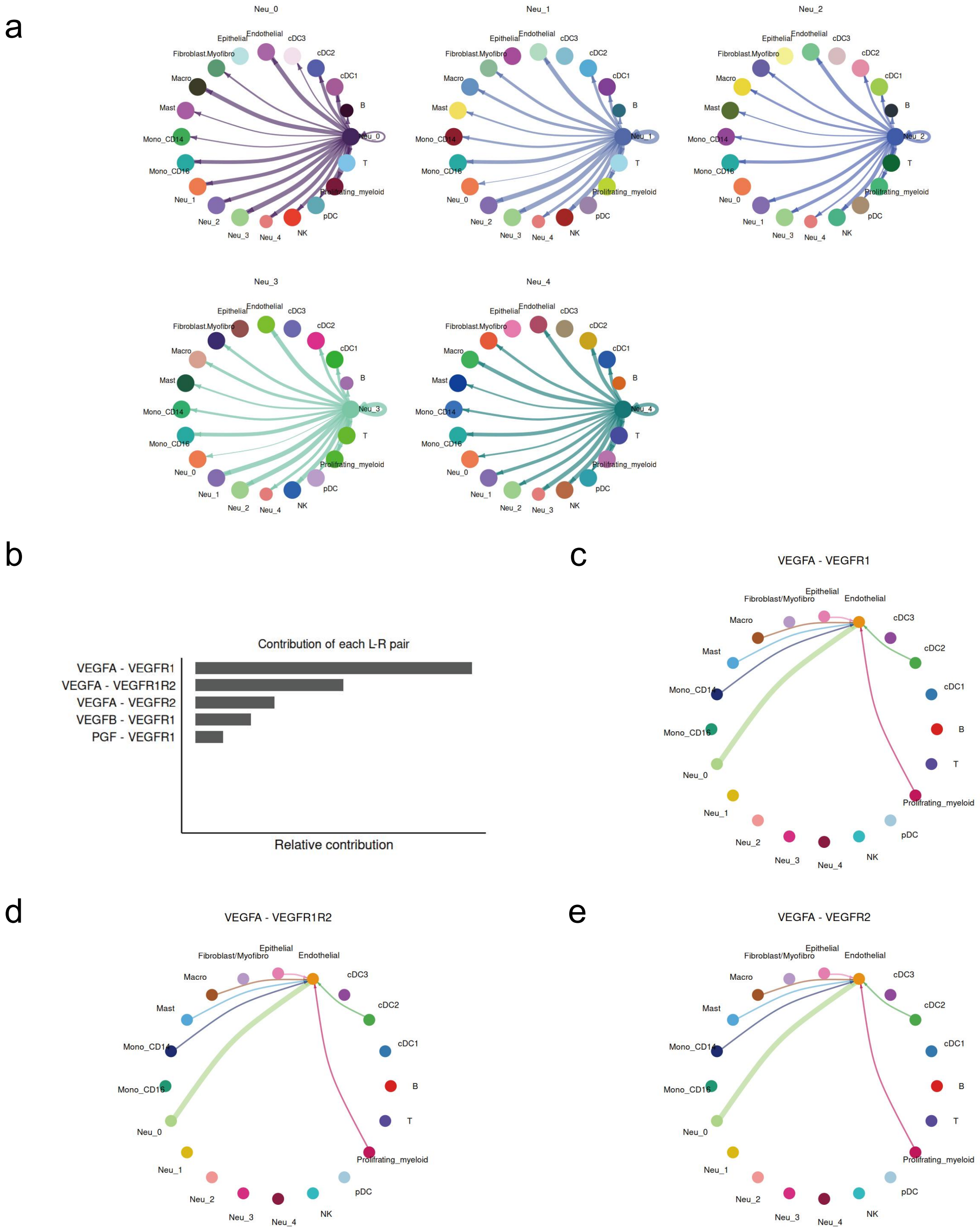
a
Neu_0
Neu_1
Neu_2
Neu_3
Neu_4
Epithelial
Endothelial
cDC3
cDC2
cDC1
B
T
Fibroblast.Myofibro
Macro
Mast
Mono_CD14
Mono_CD16
Proliferating_myeloid
pDC
NK
b
Contribution of each L-R pair
VEGFA - VEGFR1
VEGFA - VEGFR1R2
VEGFA - VEGFR2
VEGFB - VEGFR1
PGF - VEGFR1
Relative contribution
c
VEGFA - VEGFR1
d
VEGFA - VEGFR1R2
e
VEGFA - VEGFR2
Fibroblast/Myofibro
